# Stage and tissue expression patterns of *Schistosoma mansoni* venom allergen-like proteins SmVAL 4, 13, 16 and 24

**DOI:** 10.1186/s13071-017-2144-2

**Published:** 2017-05-08

**Authors:** Rafaela Sachetto Fernandes, Tereza Cristina Barbosa, Mayra Mara Ferrari Barbosa, Patrícia Aoki Miyasato, Eliana Nakano, Luciana Cezar Cerqueira Leite, Leonardo Paiva Farias

**Affiliations:** 1 Centro de Biotecnologia, Instituto Butantan, Av. Vital Brasil, 1500 São Paulo, SP Brazil; 2 Programa de Pós-Graduação Interunidades em Biotecnologia, Universidade de São Paulo, São Paulo, SP Brazil; 3 Laboratório de Parasitologia, Instituto Butantan, Av. Vital Brasil, 1500 São Paulo, SP Brazil; 4 Present Address: Instituto Gonçalo Moniz, Fundação Oswaldo Cruz (FIOCRUZ), Salvador, BA Brazil

**Keywords:** SmVAL, WISH, Acetabular glands, Germ ball, Oesophageal glands

## Abstract

**Background:**

*Schistosoma mansoni* venom allergen-like protein (SmVAL) is a gene family composed of 29 members divided into group 1 encoding proteins potentially secreted, and group 2 encoding intracellular components. Some members were found to be upregulated in the transition of germ ball - cercariae - day 3 schistosomula, suggesting that group 1 SmVAL proteins are associated with the invasion of the human host, although their functions are not completely established. Recently, we have described the localization of SmVAL7 (group 1) and SmVAL6 (group 2) transcripts in the oesophageal gland and in the oral and ventral suckers of adult parasites, respectively. The expression patterns of the two genes suggest that SmVAL7 protein plays a role in the blood-feeding process while SmVAL6 is associated with the parasite attachment and movement in the vasculature. In this way, searching for additional secreted SmVAL proteins that could be involved in key processes from skin penetration to the beginning of blood-feeding, we investigated the tissue localization of SmVAL4, 13, 16 and 24 by whole-mount *in situ* hybridization (WISH).

**Results:**

We report here the localization of group 1 SmVAL4 and 24 transcripts in the pre-acetabular glands of developing germ balls. Time course experiments of in vitro cultured schistosomula after cercariae transformation demonstrated that SmVAL4 protein is secreted during the first 3 h of in vitro culture, correlating with the emptying of acetabular glands as documented by confocal microscopy. In addition, the localization of SmVAL13 transcripts in adult male anterior oesophageal gland suggests that the respective protein may be involved in the first steps of the blood-feeding process. SmVAL16 was localized close to the neural ganglia and requires further investigation.

**Conclusions:**

Our findings demonstrate that SmVAL proteins have localizations that place them in strategic positions to be considered as potential vaccine candidates as some members are exposed to interaction with the immune system and may participate in key processes of mammalian invasion and parasitism establishment.

**Electronic supplementary material:**

The online version of this article (doi:10.1186/s13071-017-2144-2) contains supplementary material, which is available to authorized users.

## Background

Schistosomiasis is a chronic disease caused by trematodes of the genus *Schistosoma* that affects more than 230 million people in tropical and subtropical areas with more than 800 million individuals living at risk of infection [1, 2]. Transmission occurs through human contact with water containing cercariae, the infective larval stage. After penetrating the skin layer, cercariae transform into schistosomula (skin stage), which after ~72 h invade a blood or lymphatic vessel and migrate to the lungs (lung stage) where they mature for ~7 days and finally exit through a pulmonary blood vessel to reach the hepatic portal system; they then develop into adult worms and start the blood-feeding process. After pairing, the onset of egg deposition in the intestinal lumen leads to a range of morbidities in the host, such as granulomatous inflammation and periportal fibrosis [3, 4]. A fraction of the eggs is eliminated with the excreta, reaching the freshwater supply, where the miracidia larvae hatch and infect snails of the genus *Biomphalaria*. The miracidia transform into a mother sporocyst, in which daughter sporocysts produce cercarial embryos called germ balls that present several different development stages [5, 6]. Finally, mature cercariae leave the snail and enter freshwater, restarting the life-cycle.

Although the use of chemotherapy as the current strategy to control disease is effective in reducing worm burden, it is not efficient in preventing transmission and reinfection of individuals living in endemic areas. The morbidity associated with infection causes an annual loss of approximately 4.5 million disability adjusted life years (DALYs) and a recent meta-analysis suggested that these numbers should be increased from 0.5 to 15% [7]. Moreover, the possibility of selecting drug-resistant parasites must be considered when using mass treatment for long periods of time [8–10]. Therefore, the search for new intervention targets (for drugs and vaccines) remains an important goal.

In the case of helminths, one possible rationale to design a vaccine is to select secreted molecules aiming to interfere with parasite migration by blocking or impairing key processes, e.g. skin penetration, blood vessel penetration or blood-feeding. This approach is being applied for the helminth *Necator americanus*, by targeting two enzymes (Na-APR-1 and Na-GST-1), both secreted by the gut of the hookworm, intending to starve the organism [11].

In schistosomiasis, cercarial proteins released into the skin should be the first proteins accessible to the immune system and thus could be considered potential vaccine candidates. Moreover, passive immunization of mice with antisera to these secretions confers around 50% protection against challenge infection [12]. In addition, proteins secreted by adult worms during blood-feeding processes could also be interesting targets for a vaccine, since they are released into the bloodstream and carried to the liver where they can interact with antigen-presenting cells [13].

Advances in proteomics and microarray studies after the publication of the transcriptome [14] and the genome of *S. mansoni* [15], allowed the search and identification of new secreted vaccine candidates. A new gene family with similarity to venom allergens (SmVAL) was first identified by a transcriptome analysis [14], and subsequently characterized as being comprised of 29 members (SmVAL1–29), of which 23 have a secretion signal-peptide with several presenting stage specific expression [16].

A microarray study comparing gene expression in germ balls, cercariae and day 3 schistosomula, described SmVAL4, 18, 19, 20 and 24 as upregulated in germ balls; SmVAL1, 2, 16, 17 and 21 as peaking at the cercariae stage; and finally SmVAL7 and 13 as being more expressed in day 3 schistosomula [17]. Recently, we have described the localization of SmVAL6 in the suckers; and the conspicuous localization of SmVAL7 in the oesophageal gland, suggesting that the latter protein may play a role in the blood-feeding process [18].

Herein we investigated the tissue localization of SmVAL4, 13, 16 and 24 by whole-mount *in situ* hybridization (WISH). Our aim was to establish an atlas of tissue localization for SmVAL transcripts involved in key processes for immune intervention. The targets were secreted proteins going from cercariae penetration to the beginning of blood-feeding by adult worms. The criteria were to choose members from group 1 (SmVAL4 and 24) and group 2 (SmVAL 13 and 16) with high expression levels in parasite stages that could interact with the definitive host [16, 17, 19]. We also provide additional information on the SmVAL6 transcript previously localized by Rofatto et al. [18]. Our data contribute to pinpoint molecules that could be considered for further investigation as vaccine candidates.

## Methods

### Parasite material

*Schistosoma mansoni* (BH strain) cercariae were obtained via exposure of infected *Biomphalaria glabrata* snails to bright light. Schistosomula were obtained by the mechanical transformation of cercariae as previously described in [20] followed by in vitro cultivation for 3 h and 3, 5 and 7 days, using a modified protocol described by Basch [21]. Adult worms were obtained from infected hamsters by portal perfusion at 3, 5 and 7 weeks after infection with 300 cercariae using RPMI-1640 medium buffered with 10 mM HEPES (Invitrogen, Paisley, UK) and 500 units/l of heparin. Snails, infected with 40 miracidia for 35–37 days, were carefully dissected in 50% phosphate-saline buffer (PBS) and germ ball stages were immediately fixed in 4% paraformaldehyde for 16 h at 4 °C. Parasites were washed twice in PBS and then stored at 4 °C until use.

### Isolation of the anterior region of adult worms

Adult worms were extensively washed in RPMI-1640 buffered in 10 mM Hepes (Invitrogen, Paisley, UK) and 500 units/l of heparin. After the removal of any tissue debris and the exclusion of any damaged specimens under a dissecting microscope, the remaining parasites were instantly fixed by immersion in RNAlater (Invitrogen). The anterior regions of approximately 200 males were then detached just below the ventral sucker using a 26 G× ½ needle. These anterior regions, as well as the posterior regions, were maintained in RNAlater at -80 °C until use.

### Real-time PCR

Total RNA was first extracted from germ balls, cercariae, day 3 and day 7 schistosomula and adult worms (anterior and posterior regions) using TRIzol (Life Technologies), then quantified by spectrophotometry (NanoDrop 1000, Thermo Fischer Scientific) and analyzed for quality using an Agilent 2100 Bioanalyzer. After cDNA synthesis using a ThermoScript RT-PCR System (Invitrogen), RT-PCR was performed with random hexamers. Quantitative real-time PCR (qRT-PCR) reactions were performed in triplicate using SYBR Green (Life Technologies) and specific primers designed using Primer Express software (Applied Biosystems) (Additional file 1: Table S1). The transcript abundance of SmVAL7, 13 and 16 was quantified relative to actin (Smp_161930) for adult worms, while for germ ball, cercariae, day 3 and day 7 schistosomula the transcript abundance of SmVAL4 and 24 was quantified relative to 18S ribosomal subunit (sma.18 s.1). For negative controls, reactions without templates were performed for each primer set. The amplification efficiency (E) of each primer set was determined as previously described [22] using LinReg software. For each quantitative real-time PCR reaction, a normalized SmVAL expression ratio was calculated using the following equation: Ratio = (E_Ref_)^CtRef^/(E_SmVALx_)^CtSmVALx^, where E_Ref_ is the amplification efficiency of the reference gene and E_SmVALx_ is the amplification efficiency of the target gene (SmVAL) [23].

### SmVAL transcript localization by WISH

The protocols used for fixation, permeabilization, *in situ* hybridization and staining of germ balls were described by Parker-Manuel [24] and the protocols employed for cercariae and adult worms were previously described in [25]. Specific antisense RNA probes were synthesized with digoxigenin (DIG) or fluorescein in vitro using T7 or Sp6 RNA polymerase (Promega, Madison, USA) from cDNA sequences previously cloned in pGEM-T easy vector (Additional file 1: Table S1). SmVAL4 and 13 DIG-labeled sense probes were used as negative controls, while SmVAL7 DIG-labeled probe was used as a positive control. Two different protocols were applied for germ balls and adult worms as follows.

Briefly, germ balls were fixed in 4% paraformaldehyde and permeabilized via dehydration in PBS-T solutions (PBS 0.1% Tween 20) containing 25, 50 and 75% methanol at room temperature. After rehydration, germ balls were first incubated in pre-hybridization buffer (50% formamide, 5× SSC (pH 7), 2% BMB, 1% Triton X-100, 0.5% CHAPS, 1 mg/ml yeast RNA, 50 μM EDTA and 50 μg/ml heparin) for 1 h at 65 °C, followed by a new incubation in fresh buffer containing 2 μl/ml of DIG-labeled probes SmVAL4, 24 or the control SmVAL4 sense probe for 16 h at 65 °C under constant rotation. Parasites were then washed in TBST (0.14 M NaCl, 2.7 mM KCl, 25 mM Tris-HCl pH 7.5, 0.1% Tween-20) and blocked for 90 min in TBST with 10% inactivated sheep serum. After blocking, germ balls were incubated in fresh blocking solution with anti-DIG antibody conjugated with alkaline-phosphatase (1:2000) for 16 h at 4 °C. After several washes, BM Purple (Roche, Basel, Switzerland) was used as enzyme substrate to reveal the transcript localization. Images were captured using a Nikon Eclipse E200 microscope coupled to a Microscope Eye-Piece Camera (Dino-Lite, Taiwan).

Adult worms were first fixed in Carnoy (ethanol: chloroform: acetic acid, 6:3:1% v/v) for 2 h at 4 °C and then fixed in MEMFA (0.1 M MOPS, 2 mM EGTA, 1 mM MgSO_4_, 3.7% formaldehyde in H_2_O) for 1 h. Parasites were first rehydrated, permeabilized by proteinase K (10 μg/ml in PBST) treatment for 28 min and then washed twice in 0.1 M of triethanolamine (pH 7.8) diluted in PBS. After two washes in triethanolamine solution containing acetic anhydride (0.25%) and two washes in PBS, the worms were refixed in 10% formalin and then incubated with hybridization buffer (50% formamide, 5× SSC (pH 7), 100 μg/ml heparin, 1× Denharts, 0.1% Tween-20, 0.1% CHAPS and 10 mM EDTA) containing yeast RNA (1 mg/ml) and SmVAL DIG-labeled probes for 16 h at 60 °C. To block non-specific binding, samples were pre-incubated in MAB (100 mM maleic acid, 150 mM NaCl, 0.1% Tween-20, pH 7.8) containing 2% BMB (10% maleic acid) and 20% inactivated sheep serum for 2 h at room temperature. Next, all samples were incubated with fresh blocking solution containing anti-DIG antibody conjugated with alkaline-phosphatase (1:2000) for 16 h at 4 °C. After several washes in Alkaline-phosphatase Buffer (100 mM Tris pH 9.5, 50 mM MgCl_2_, 100 mM NaCl, 0.1% Tween-20), the color was developed using BM Purple (Roche), INT/BCIP (Roche) or Fast Red (Sigma-Aldrich) as enzyme substrates, after which the parasites were monitored to detect sites of gene expression and images were captured as described for germ balls. SmVAL13 DIG-labeled sense probe was used as a negative control.

### Double localization of SmVAL13 and SmVAL7 transcripts

For double *in situ* localization of SmVAL13 and 7 transcripts in adult worms, parasites were initially hybridized with SmVAL13 DIG-labeled probe and SmVAL7 Fluorescein-labeled probe simultaneously. These were then incubated with anti-fluorescein antibody and the first color development step was performed using INT/BCIP (Roche) as an alkaline-phosphatase substrate for SmVAL7 transcript visualization (orange). Next, the parasites were washed several times in alkaline phosphatase buffer to remove any remaining substrate, after which the alkaline phosphatase was inactivated by fixing the parasites for 20 min in 4% formaldehyde/PBST, followed by 5× rinsing in PBST and a 30-min incubation period at 65 °C. The parasites were subsequently re-blocked for 2 h at room temperature and then incubated with anti-DIG antibody. The color was developed using BM Purple (Roche) as enzyme substrate for SmVAL13 transcript visualization (purple) and images were captured as described for germ balls. SmVAL13 DIG-labeled sense probe was used as negative control.

### Processing of cercaria to day 7 schistosomula and their secretions

Parasites and their released proteins (RP) were collected in a time course manner after cercariae mechanical transformation process [20], and after culturing schistosomula for 3 h and 3, 5 and 7 days [21]. Briefly, ~170,000 cercariae were concentrated after 30 min incubation in an ice bath followed by 3 min centrifugation at 2000× *g* at 4 °C. The tail loss was obtained by resuspending packed cercariae in 8 ml of RPMI 1640 (GIBCO) at 37 °C and vortexing for 90 s in a Vortex mixer [20]. Then, parasites were allowed to decant for 10 min and the tail-rich supernatant was collected and centrifuged for 3 min at 200× *g* to separate the medium containing the cercarial released proteins (named Cerc RP) from the pellet containing the tails. Cercarial bodies were then resuspended in a further 8 ml of medium and incubated at 37 °C for 3 h under a 5% CO_2_ atmosphere, the released proteins (named 3-h RP) were collected in the medium supernatant after centrifugation (3 min at 200× *g*). The pellet containing the parasites were then washed (5–6 times) to remove any remaining tails, through the resuspension in 8 ml of RPMI 1640 (GIBCO) followed by parasite decantation for 5 min and discard of the supernatant containing the tails. Schistosomula (~80,000) were then resuspended in 8 ml M-169 medium and cultured for 7 days [21]; parasite viability was measured by microscopic observation of movements (contractions) and established to be around 85% until the 7th day of in vitro culture.

The parasites and the culture media (7.5 ml) corresponding to parasite-released proteins (named 3, 5 and 7-day RP) were carefully collected after centrifugation at 200× *g* for 5 min [26, 27], leaving approximately 0.5 ml of the volume above the pellet to avoid pipetting any parasite debris. The supernatant containing secretions (RP) was stored at −20 °C after addition of 100 μl of 10× Protease Inhibitor Cocktail (Sigma-Aldrich). The secreted proteins were TCA precipitated for concentration and resuspended in 1/100 of the initial volume with 40 mM Tris (pH 7.4), 2% SDS plus Protease Inhibitor Cocktail (Sigma-Aldrich) and 20 μl of each sample were used in SDS-PAGE. Parasites were also collected, washed in PBS to eliminate any remaining secreted protein or dead parasites, and total protein extracts were prepared in the same buffer by sonication (4 cycles of 2 min with pulses of 0.75 s, 40% amplitude). Samples were centrifuged at 20,000 × *g* for 30 min at 4 °C and the supernatant was recovered and used for the western blot assays. Total protein concentration was determined by Lowry’s method (DC Protein Assay Bio-Rad) using bovine serum albumin as a standard. Western blots were performed as previously described [28] using 10 μg of parasite extracts, 20 μl of concentrated secreted proteins (100×), recombinant SmVAL4 protein as a positive control and anti-rSmVAL4 (1:2000) polyclonal antibodies.

### Acetabular gland emptying

To observe the course of acetabular gland development and emptying in parasites cultivated in vitro, Alexa Fluor 647-conjugated lectin (PNA) was used as previously described [29]. First, *ex-vivo* germ balls, shaded cercariae and cultured schistosomula (collected at either 3 h or 3, 5 or 7 days) were fixed in 4% paraformaldehyde for 16 h at 4 °C, washed twice in PBS and stored at 4 °C until use. These were then permeabilized in permeabilizing buffer solution (PBS, 1% Triton X-100, 0.1% SDS, 10% goat serum, 0.1% NaN_3_) for 30 min under rotation at 4 °C. Parasites were then incubated for 16 h at 4 °C in antibody diluent solution (PBS, 0.3% Triton X-100, 0.05% Tween-20, 10% goat serum) containing FITC-phalloidin (1 μg/ml) for musculature staining and Alexa Fluor 647-conjugated PNA (1:250 dilution from a 1 mg/ml solution) for glycoprotein visualization. Parasites were washed three times in PBS at 4 °C, mounted on slides using ProLong Gold Antifade Mountant (ThermoFischer Scientific) and images were captured using a Zeiss Axiovert 100 microscope linked to a Zeiss LSM 510 Meta confocal system.

## Results

### Transcriptional profiles of SmVAL4, 6, 7, 13, 16 and 24 at different parasite stages

**Fig. 1:**
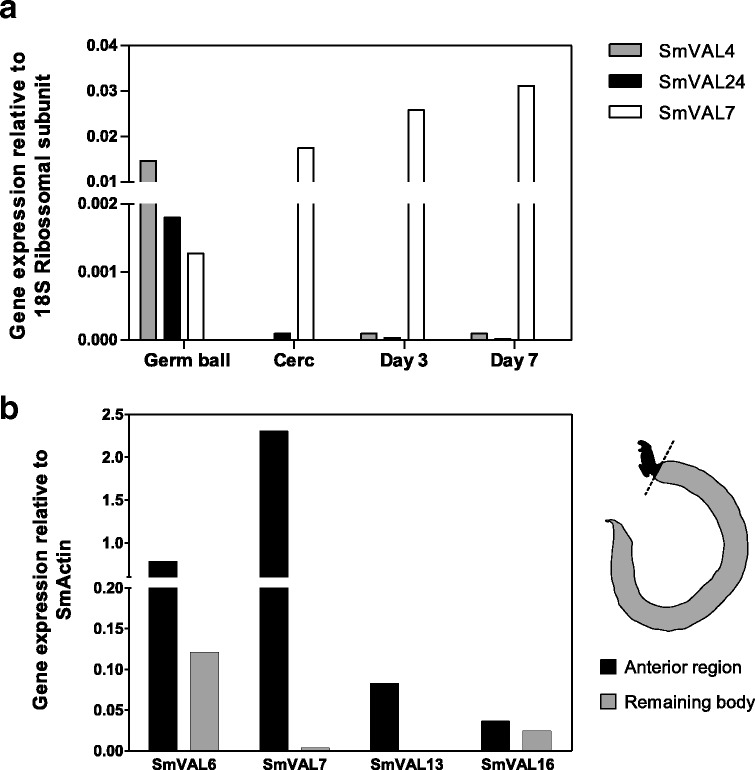
Transcriptional profiles of SmVAL4, 6, 7, 13, 16 and 24 at different parasite stages. **a** Relative expression of SmVAL4, SmVAL7 and SmVAL24 genes in germ balls, cercariae, day 3 and day 7 schistosomula. Data were normalized according to 18S ribosomal subunit expression. **b** Relative expression of SmVAL6, 7, 13 and 16 in the anterior region in adult males in comparison to the remainder parasite body. The data were normalized according to SmActin expression. SmVAL7 was used as a positive control. The schematic drawing illustrates where the parasites were cut to separate the anterior region from the remainder body

Concerning group 1 SmVALs, those likely to be secreted/excreted, we compared the gene expression levels of SmVAL4 and 24 by qRT-PCR at germ ball, cercariae, as well as both day 3 and day 7 schistosomula stages. Our data showed upregulation of these genes at the germ ball stage, in relation to cercariae, day 3 and day 7 schistosomula. SmVAL7 was used as a control, since its expression is highly upregulated in the later schistosomula stages (Fig. 1a).

We next investigated the transcriptional levels of the group 2 SmVAL members 6, 13 and 16, following microdissection of the entire oesophageal region and the matching posterior regions of adult male worms. We also analyzed group 1 SmVAL7 expression levels as a positive control since its transcript was previously localized in the oesophageal gland of adult worms [18]. Our qRT-PCR analysis showed that the SmVAL7 and SmVAL13 genes were exclusively expressed in the adult male anterior region in comparison to the rest of the parasite body. On the other hand, the expression of SmVAL6 and SmVAL16 were more balanced, with slightly increased expression observed in the anterior compared to posterior region (Fig. 1b). These data were used to guide us with respect to the parasites stages in which to conduct WISH assays and the parasite regions to be further investigated.

### SmVAL4 and 24 transcripts are localized in the pre-acetabular gland of immature cercariae

**Fig. 2:**
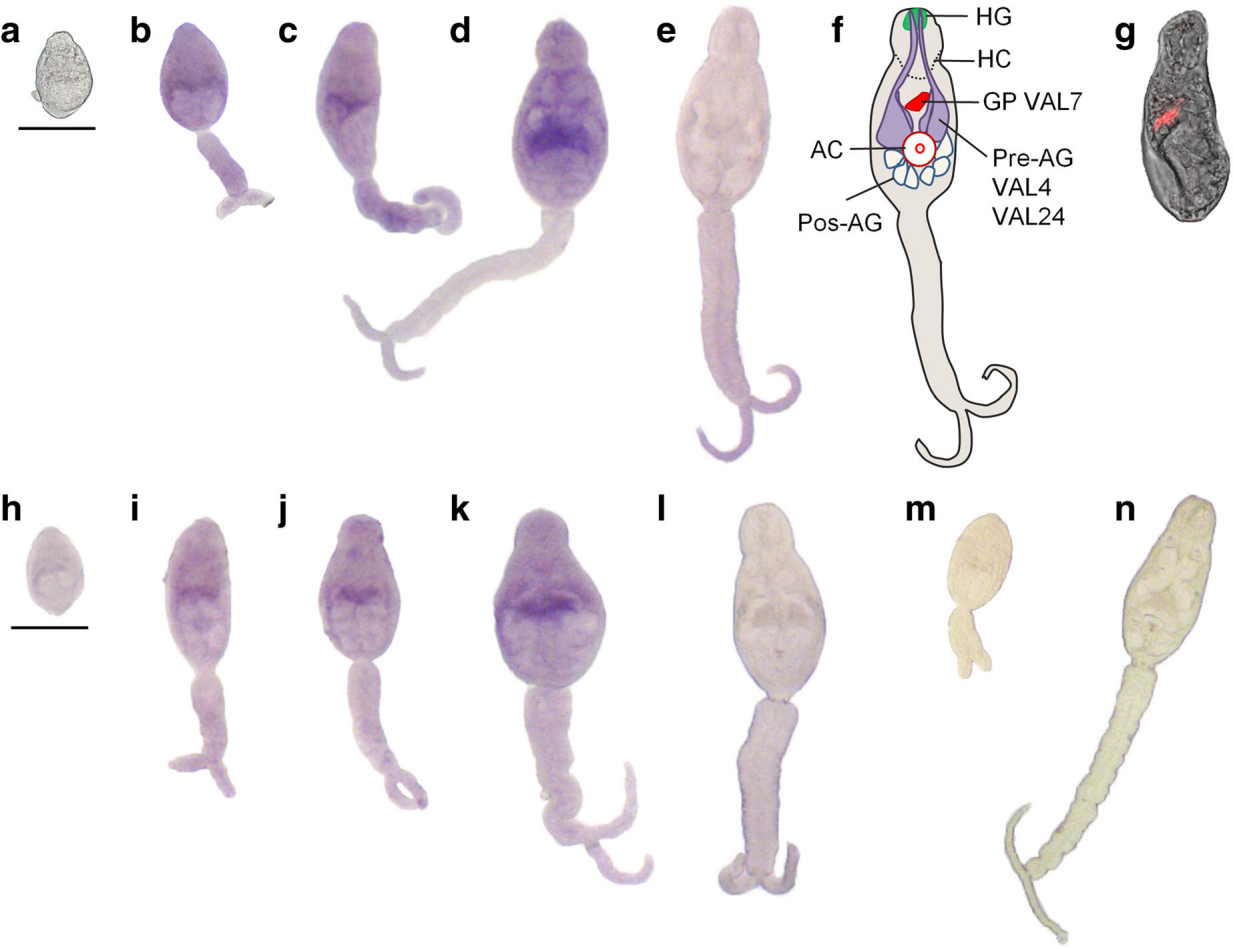
SmVAL4 and 24 transcripts localization in the pre-acetabular glands of germ ball stages by WISH. Germ ball stages of *S. mansoni* were analyzed using SmVAL4 (**a**-**e**) and SmVAL24 (**h**-**l**) DIG-labeled probes in the following stages: small round germ ball (**a**, **h**); stubby tailed stage (**b**, **i**); elongating immature stage (**c**, **j**); immature cercariae (**d**, **k**) and mature cercariae (**e**, **l**). The schematic drawing of a cercaria shows the two pairs of pre-acetabular glands with acetabular ducts (Pre-AG), the three pairs of post-acetabular glands (Pos-AG), the acetabulum (AC), the head capsule (HC), the gut primordium (GP) and the head gland (HG) (**f**). SmVAL7 probe was used for gut primordium visualization in mature cercariae using fast red substrate and confocal microscopy (**g**) as previously described [18]. SmVAL4 sense DIG-labeled probe was used as a negative control (**m**, **n**). Color was developed for 90 min. *Scale-bars*: 50 μm

After establishing that SmVAL4 and SmVAL24 gene expression peaked at the germ ball stage, we investigated the tissue localization of these transcripts by WISH. Detectable levels of these transcripts were seen in the ‘stubby-tailed’ and ‘elongating-tail’ stage prior to cercarial maturation (Fig. 2b–d, i–k), but not in the small round germ balls (Fig. 2a, h). Marked signals were detected in immature cercariae, as can be observed by a band across the middle of the body along the anterior and lateral edges of the pre-acetabular glands (Fig. 2d, k). It is interesting to note that no signal was observed in the mature cercaria stage, even after extending the time of color development (Fig. 2e, l). We used SmVAL7 probe as a positive control for a known gene expressed in mature cercariae, which revealed the gut primordium (GP) as previously described [18], note that cercarial tail was detached during processing (Fig. 2g). We also tried to localize SmVAL16 in the cercariae stage, but no signal was observed for this transcript, even after prolonged time of color development (data not shown).

### SmVAL4 protein is secreted during the first hours of in vitro culture

**Fig. 3:**
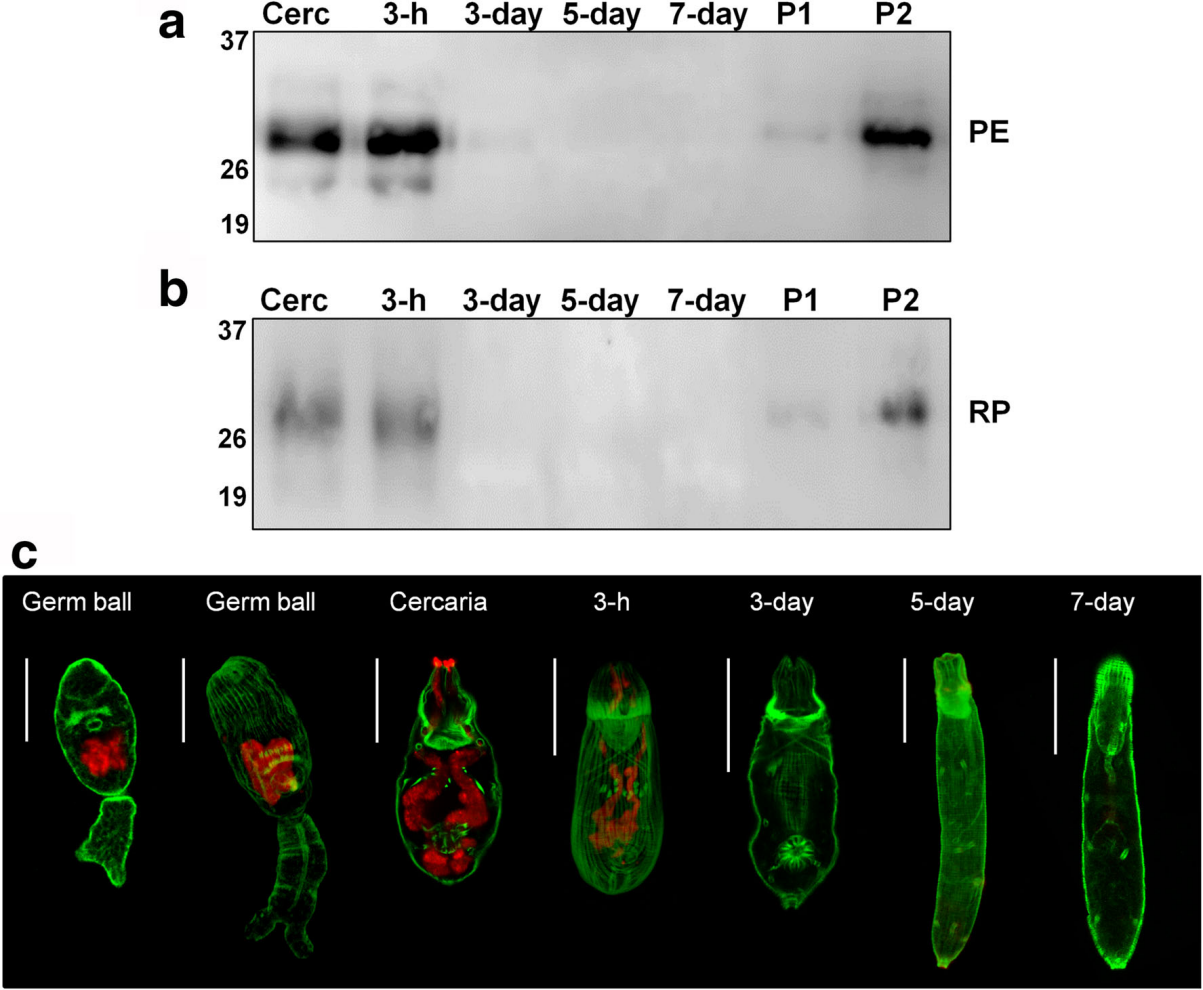
Identification of in vitro secreted SmVAL4 and visualization of PNA-stained acetabular glands of parasites cultured for up to 7 days. Polyclonal anti-rSmVAL4 was used to identify native SmVAL4 in total parasite extracts (PE) and secretions containing released proteins (RP) of cercariae (Cerc), 3-hour schistosomula (3-h), day 3 schistosomula (3-day), day 5 schistosomula (5-day), and day 7 schistosomula (7-day). P1 and P2 - positive controls, recombinant SmVAL4, 15 and 50 ng, respectively. For PE, 10 μg of protein extract was applied in each lane **a**. Secretions derived from ~80.000 cultured schistosomula (20 μl) were used in **b**. Molecular weight (kDa) is marked on the left side of the images. **c** Parasite stages stained with Alexa-fluor 647-conjugated PNA (*red*). *Green* indicates FITC-phalloidin staining muscle fibers containing actin. *Scale-bars*: 50 μm

We have previously demonstrated that SmVAL4 is present in cercariae extract and secreted during the first hours after mechanical transformation in vitro [28]. Herein, we extended the time course of analysis to seven days after transformation, conducting further assessment of parasite extracts and the proteins released (Fig. 3a, b). Consistent with our previous data, significantly higher levels of this protein were secreted by 3-h schistosomula, which corresponds to acetabular glands content release. No signal of this protein was detected in secretions of schistosomula from day 3 up to day 7 stage (Fig. 3b). To validate the emptying of the acetabular gland content in these in vitro cultivated parasites, we labeled parasites with PNA lectin, known to interact with glycoproteins present in the acetabular glands. Positivity was seen in both pre- and post-acetabular glands at later germ ball development stages, as well as in cercariae and 3-h schistosomula. Acetabular gland content had already been completely emptied in day 3-old parasites, as evidenced by the absence of signal at this stage, as well as in day 5 and day 7 schistosomula (Fig. 3c).

### SmVAL6, 13 and 16 transcripts localization in the anterior region of adult worms

Differential expression of SmVAL6, 7, 13 and 16 genes was detected by qRT-PCR in adult male anterior region in comparison to the rest of the parasite body. The anterior region of the parasite contains different structures and cell types, such as the oral and ventral suckers, the neural ganglia, cell bodies of the tegument and cell bodies of the two oesophageal gland compartments. The WISH methodology was used to investigate the expression of SmVAL6, 7, 13 and 16 within these structures.

**Fig. 4:**
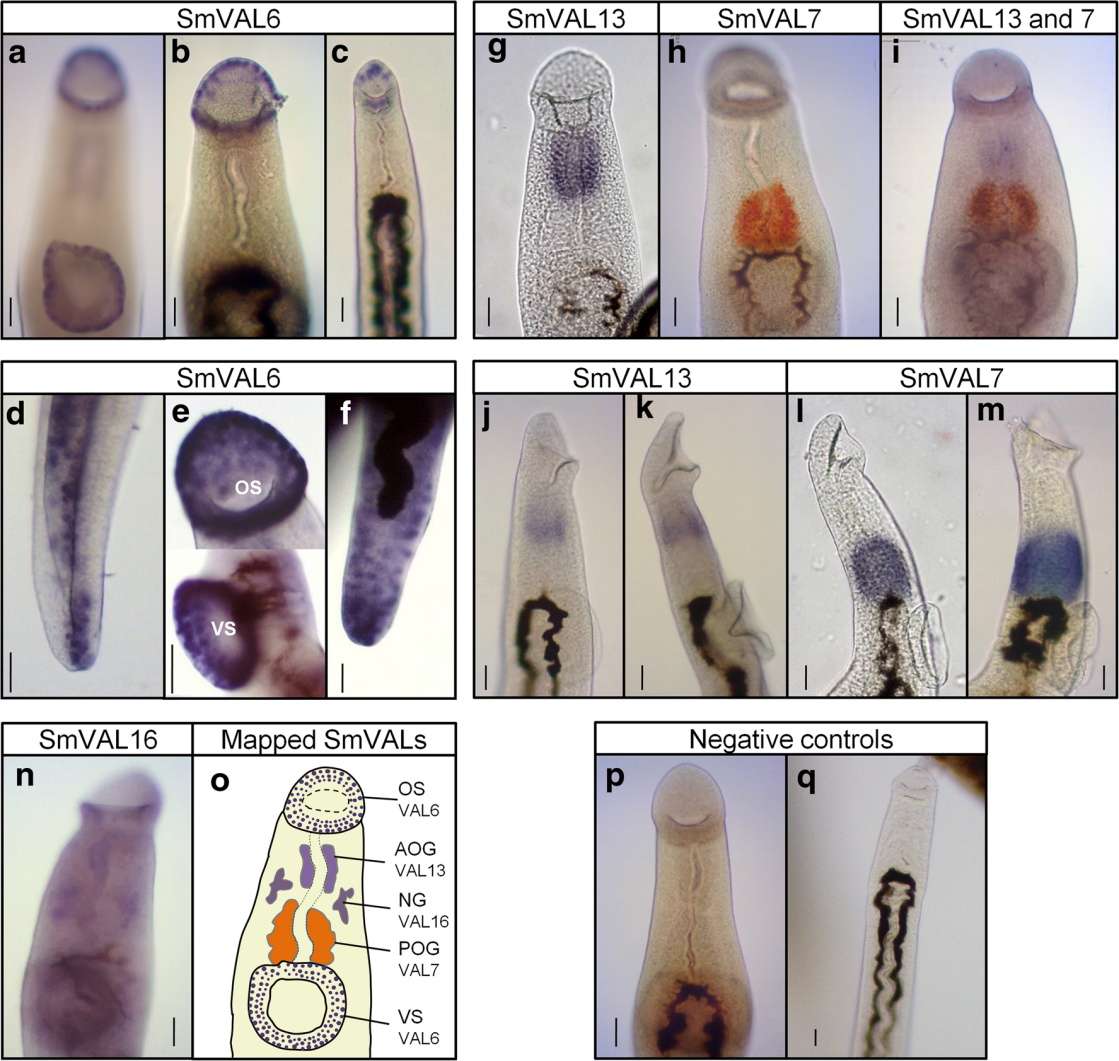
Localization of SmVAL6, 7, 13 and 16 in the anterior region of adult *Schistosoma mansoni* by WISH. Adult worms were analyzed using SmVAL6, 7, 13 and 16 DIG or fluorescein-labeled probes and revealed with two different substrates (BM Purple - *purple*, or INT/BCIP - *orange*). SmVAL6 transcripts identified in the oral and ventral suckers of adult male (**a**, **b**, **e**) and female worms (**c**) and in the tegument cell bodies of the posterior region of the parasite (**d**, **f**). SmVAL13 localization in the anterior oesophageal gland of 7-week-old male adult worms (**g**), as well as, in 3 and 5-week-old worms (**j**, **k**). SmVAL7 localization in the posterior oesophageal gland of 7-week-old male adult worms, revealed with INT/BCIP (*orange*) (**h**), as well as, in 3 and 5-week-old worms (**l**, **m**). Double *in situ* localization of SmVAL13 (*purple*) and SmVAL7 (*orange*) in the anterior and posterior oesophageal glands, respectively (**i**). SmVAL16 transcript localized close to the neural ganglia of male adult worm (**n**). No staining was observed in the tissues of male and female worms hybridized with the negative control (SmVAL13 sense probe) (**p**, **q**), respectively. Schematic representation of SmVALs mapped so far (**o**): OS, oral sucker; VS, ventral sucker; AOG, anterior oesophageal gland; POG, posterior oesophageal gland; NG, neural ganglia. Development of color was observed after approximately 4 h of the enzyme reaction for SmVAL7 and 13, 72 h for SmVAL16 and up to 12 h for SmVAL6. *Scale-bars*: 50 μm

Previously, SmVAL6 was found to be expressed in the oral and ventral suckers of male worms [18], and, herein, we confirm this finding and provide additional information by increasing the time for color development after substrate addition. The first signal detected (after 2 h) was very conspicuous in the ventral and oral suckers of adult male worms. It is possible to observe individually stained cells throughout different layers of the suckers (Fig. 4a, b, e). After 3 h, staining in cells along the parasite body becomes evident, which appear to be cell bodies of the tegument (Fig. 4d). Concerning female worms, the level of SmVAL6 expression was found to be much lower, similar to previous reports [16, 18]. However, by extending the time for color development to 6 h, a signal became observable in the oral sucker of female worms (Fig. 4c). This was later followed by the detection of some cells in the posterior region of the female parasite bodies (with ~10 to 12 h) (Fig. 4f).

Regarding SmVAL13 localization, our results using WISH showed that the SmVAL13 transcript was localized in the anterior oesophagus of male worms, a dense cluster of gland cells present in the first half of the oesophagus (Fig. 4g). No staining was observed in any tissues of 7-week-old female adult worms (data not shown). SmVAL7 expression in the posterior oesophageal gland cell bodies was used as a positive control (Fig. 4h). In order to provide a clear distinction between the anterior and posterior oesophageal glands, as well as to show the localization of SmVAL13 and 7 transcripts within the same specimen, we performed a double *in situ* hybridization using a SmVAL13 DIG-labeled probe (purple) and a SmVAL7 Fluorescein-labeled probe (orange) (Fig. 4i). Furthermore, we also analyzed the earlier stages, in which adult worms begin to reach the hepatic portal system and feed on erythrocytes (3-week worms) and start the oviposition, demanding energy to produce eggs (5-week worms). At these earlier time points, SmVAL13 (Fig. 4j, k) and SmVAL7 (Fig. 4l, m) expression was detected in the anterior and posterior oesophagus, respectively.

The SmVAL16 probe produced the weakest signal observed and was only detected after very long time of color development (72 h). The signal appeared between the anterior and posterior oesophagus, but was distant from the oesophageal cell bodies, i.e. positioned close to the neural ganglia (Fig. 4n). It is also possible to observe the precipitation of some substrate in the esophagus lumen due to the extended time of color development, but not in the oesophageal glands. No specific staining was observed in the remainder of the body of male worms, nor in any tissue of female adult worms (data not shown). A summary of the anterior regions where all the SmVALs were mapped is highlighted in Fig. 4o. No staining was observed in any tissue of the male and female adult worms probed with the SmVAL13 DIG-labeled sense probe used as a negative control (Fig. 4p, q).

## Discussion

The literature contains divergent results with respect to peak expression of some members of the group 1 SmVALs. Previous qRT-PCR data on SmVAL4 expression showed upregulation during the cercariae stage [16], while microarray data showed that SmVAL4 and 24 expression was upregulated in germ balls in relation to cercariae and day 3 schistosomula [17]. To clarify these divergences and pinpoint the most appropriate stages for subsequent *in situ* hybridization, we performed a comprehensive comparative study of the transcriptional levels in germ balls, cercaria, day 3 and day 7 schistosomula. Our data revealed SmVAL4 and SmVAL24 transcripts to be upregulated in germ balls, which stands in agreement with data reported by Parker-Manuel et al. [17]. In contrast, Chalmers et al. [16] showed upregulation of SmVAL4 in cercariae in comparison to the mother sporocyst and 13 other parasite stages [16]. However, these authors obtained mother sporocysts by in vitro transformation, which does not directly correspond to *ex-vivo* dissected germ balls, which may account for these divergent results.

SmVAL4 and SmVAL24 transcripts were found to be localized in the pre-acetabular glands of germ balls by *in situ* hybridization, which is consistent with the upregulation detected by qRT-PCR. Moreover, the intensity of the detected signal corroborates the estimated abundance of mRNA measured by qRT-PCR. These results emphasize the importance of accurate determination of the transcriptional profiles of stage-associated genes in order to guide tissue localization by WISH in schistosomes.

It is possible that some of the “released” proteins secreted by cercariae, could be originated from the artificial mechanical transformation process (ice incubation followed by vortexing) that could be harsher than the natural transformation process. SmKK7, for example, was first described as a secreted putative potassium channel blocker present in secretions from mechanically transformed cercariae [26]. The protein was later revealed to be present in the ciliated sensory endings (reviewed in [30]) rather than in acetabular glands. Thus, another important contribution of our work was the establishment of the precise site of gene expression for proteins that have been previously detected by high throughput proteomic studies of cercarial secretions.

With respect to acetabular function, three pairs of post-acetabular glands, positioned posterior to the acetabulum [31], produce mucins responsible for the adhesion of cercariae to the skin surface, while the two pairs of pre-acetabular glands situated anterior to the acetabulum, contain a variety of well-characterized proteolytic enzymes that assist in skin penetration (reviewed in [13]). The majority of these proteolytic enzymes are metallo and serine proteases, the former also known as cercarial elastases, whose transcripts are expressed in the germ ball (sporocyst stage) [17, 32]. Thus, as SmVAL4 and SmVAL24 transcripts were found to be localized in the pre-acetabular glands, it is possible that their respective proteins could play an active role in invasion of the mammalian host, with transcription peaking in germ ball stages and the protein being expressed and released by cercariae.

Although the molecular weight of the protein detected by anti-SmVAL4 antibody, is consistent with the SmVAL4 protein, we cannot completely exclude the existence of potential cross-reactivity with other secreted SmVALs, such as SmVAL10 and 18, which have also been identified in cercarial secretions [26]. In addition, the recent localization of SjVAL1 from *S. japonicum* in the head gland by Chen et al. [33] raises the hypothesis that some SmVALs could be expressed in the head gland of *S. mansoni*, a structure containing lytic secretions which are thought to be used by day 3 schistosomula to penetrate blood vessels. Moreover, the SmVAL4 protein was recently identified by proteomics in the tegument of schistosomula after culturing for 3 h, 2 and 5 days [34]. However, our WISH data provided here do not support the expression of SmVAL4 and SmVAL24 in these structures (head gland or tegument).

To date, two functional studies have associated group 1 SmVAL members with different biological processes. Recombinant SmVAL4 produced in *Pichia pastoris* was found to bind lipids such as cholesterol, and demonstrated to complement in vivo the sterol-export phenotype of yeast mutants lacking the putative ortholog protein [35]. SmVAL9, a protein secreted during miracidia to sporocyst transformation, was expressed in *E. coli* and has been found to stimulate transcription of genes involved in host extracellular matrix remodeling in vitro [36]. Thus, no clear molecular function has been defined for the family. One possibility, as proposed by Hewitson et al. [37], is that the SCP/TAPS domain operates as an adaptable protein framework facilitating the evolution of various specialized functions [37].

The involvement of the group 2 SmVALs with respect to the host interface remains unclear, since these proteins are not expected to be secreted. However, the structure of SmVAL6 and its tegument localization suggests that this protein has adapted to play some role in the host-parasite interface. In addition to its SCP/TAPS domain, it has acquired the characteristics of a Micro-Exon-Gene (MEG) in its C-terminal region [38], which produces variant proteins through alternative splicing. From this perspective, some adaptive advantage must be present in order for the parasite to sustain such a complex mechanism of gene expression and protein variation. Our previous study allowed only the delineation of SmVAL6 expression in the suckers, but we were not able to identify a specific group of cells within them [18]. Herein, the WISH data provided detailed evidence of transcript expression around the rim region and in different cell layers of the oral and ventral suckers. Additionally, by extending the time of color development, we were able to detect expression along the parasite body in the cell bodies of the tegument, which is in agreement with the previous detection of the protein in tegument fractions through mass spectrometric analysis [18]. The localization of SmVAL16 in the neural ganglia is quite puzzling, since it would be the first description of a parasitic SCP/TAPS domain protein expressed in a neural tissue. Interestingly, the distantly related GLIPR gene has been described as highly expressed in human brain cancer and glioma cell lines [39].

The SmVAL13 gene, was first depicted as an upregulated gene in female worms by qRT-PCR [16], and then subsequently identified in the anterior region of adult male worms by microarrays [40, 41], and more recently in day 3 schistosomula [17]. Herein, we provide definitive evidence that this gene is actually expressed in the anterior oesophagus of adult male worms; we could not detect SmVAL13 expression in adult female worms by WISH. Our double *in situ* hybridization showed that the SmVAL13 transcript is localized exclusively in the anterior oesophageal gland, while SmVAL7 is localized exclusively in the posterior oesophageal gland, confirming the status of these structures as two distinct glands in *S. mansoni* [42, 43]. Recently, several studies demonstrated that schistosome oesophagus is not just a conduit that connects the mouth opening with the gut, actually it initiates the processing of ingested blood before it reaches the gut lumen [43, 44]. It is divided into anterior and posterior compartments, each one required to play different roles in the blood processing. The current view is that erythrocytes are ruptured as they pass through the oesophagus, leucocytes are tethered and killed and platelets are eliminated preventing clot formation [43]. The precise function of SmVALs in this context is still unknown and we can only speculate about their role.

The localization of SmVAL13 transcript in the anterior oesophageal gland indicates that the protein could be interacting with incoming erythrocytes and leucocytes to destabilize their plasma membranes [44]. This interaction would occur in the oesophageal lumen if SmVAL13 is secreted by a non-classical secretion pathway, or even intracellularly if the protein is not secreted, during subsequent steps after lipid membranes absorption. It is important to remember that the transfer of the lipophilic dye PKH2 from labeled erythrocytes starts in the anterior compartment and is completed in the posterior [45]. Regarding SmVAL7 function, its localization in the posterior oesophagus suggests that it may help in erythrocyte lysis or leucocyte tethering and killing [44]. Noteworthy, SmVAL4 was found to bind lipids such as cholesterol [35], which is especially abundant in the plasma membrane of mammalian cells, thus it would be interesting to investigate whether SmVAL 7 and 13 present the same feature. Furthermore, several members of the SCP/TAPs protein superfamily were identified in the oesophageal glands of different nematode parasites [46–49], thus it is reasonable to expect that these genes have been evolutionarily selected to play a role in the feeding process.

Besides SmVAL13, recently, MEGs 12, 16, 17 and phospholipase A2 transcripts were identified in the anterior oesophageal gland of adult worms by WISH [50]. In the same study, a total of eleven MEGs, two lysosomal hydrolases and one glycosyltransferase were localized to the posterior oesophagus. Thus, the repertoire of proteins expressed in the oesophageal glands is much broader than a few SmVALs, which poses a challenge to pinpoint the most relevant proteins, or consider the use of a cocktail for immune intervention.

A recent study demonstrated that immunization with a cocktail of native secreted VAL proteins from *Heligmosomoides polygyrus* induced high levels of protection [51], which may suggest a similar strategy against *S. mansoni*. Thus, as a rationale to design a schistosome vaccine that blocks or impairs parasite migration and/or the blood-feeding process, it is important to characterize and investigate proteins involved in the initial steps of infection (e.g. SmVAL4 or SmVAL24, localized in pre-acetabular glands of cercariae) as well as those present in established adults (e.g. SmVAL13, localized in the anterior oesophageal gland and SmVAL7, in the posterior oesophageal gland of adult worms); experiments are currently underway to test this hypothesis.

## Conclusions

The data described herein suggests that since the localization of SmVAL4, 7, 13 and 24 has been well established, these proteins could represent interesting targets for a schistosome vaccine. Further studies should be conducted to elucidate their functions with the aim of interfering in the invasion of the mammalian host, migration through blood vessels to the lungs and with the blood-feeding process in mesenteric veins. A cocktail of these SmVAL proteins, or their combination with other strategic targets, may constitute a new road for an effective anti-schistosome vaccine.

## Additional file

Additional file 1:
**Table S1.** Set of primers/probes used to detect gene expression by qRT-PCR and WISH. (PDF 364 kb)

## Abbreviations

Ct: Cycle threshold
qRT-PCR: Quantitative real-time polymerase chain reaction
SmVAL: *Schistosoma mansoni* venom allergen-like protein
WISH: Whole-mount *in situ* hybridization
