# Supplementary material for: Stage and tissue expression patterns of Schistosoma mansoni venom allergen-like proteins SmVAL 4, 13, 16 and 24
Source: Parasit Vectors. 2017 May 8;10:223. doi: 10.1186/s13071-017-2144-2 (PMC5422958; doi:10.1186/s13071-017-2144-2)
Supplement: Additional file 1: — Table S1. Set of primers/probes used to detect gene expression by qRT-PCR and WISH. (PDF 364 kb) [file 13071_2017_2144_MOESM1_ESM.pdf]

Table S1. Set of primers/probes used to detect gene expression by qRT-PCR and WISH.

Sybr System			
Genes	*S. mansoni* Gene DB	Forward primer (5'-3')	Reverse Primer (5'-3')
SmVAL4	Smp_002070	GGAAGGACAACGAGCAATCT	CGTTTAGCTTGTTGTTTAGCTTTATT
SmVAL6	Smp_124050	CTTATCATCCAAATTCACGTCATTTT	AAAGGCCAATCCGAATCCA
SmVAL7	Smp_070240	TGGAGTCATAAATTGGCTGAAATG	CAGATGCTGCTTGTCTAACTTTAGGA
SmVAL13	Smp_124060	TGTGATGAATATGGAGAAAATTTGG	TGAATCTCATCGTACCAGTTTCGT
SmVAL16	Smp_124070	AGGATTCGGTTCTGCATTATCC	CATCTCTTGGTGGAGCAGTTATCTT
SmVAL24	Smp_141550	GAAAGAATCGAACTACTATGACCACAAT	TGCTTCAACTATCTGCTTGTAAGTGTT
18S Ribossomal	sma.18s.1	TCGGCGACGGATCTTTCA	CCGGAATCGAACCCTGATTC
SmActin	Smp_161930	CGTTGGACGACCTCGACAT	TTTGTGTAGGTTGGACGCTCTATATC

Probes Templates

SmVAL4	aagttatcggaaggacaacgagcaatctataatttccataaaaaagttcgtaaagatgtaaaaaattgcaggatacctggtcaac<br>ctccagctaaaaatctaacaaagttgaaatggaataaactattagctaataaagctaaacaacaagctaaacgatgtaaatatga<br>ttcaaatgatccaaatgattttattattggggattttgaatcaattggacaaaatttagccgattatccaacaattgaaggtgca<br>atgaaagattggttagaagagtataaaaattataattttgaaaagaatcaatgtaatggtgattgtaaaaattataaacagatgg<br>tttggaataccactgaagaaataggctgcggttatgaaaaatgtggaaagaactatttgattgtttgcaattatgcaccaggga<br>ctctgaagatagaccatatgaagctaaaccagaaagtaaatgtaataaatcagaataa
SmVAL6	atgattaatgaacgttttaatgatcaagctatacgggaacataatcgacttcgttctctacatggatgtccagaacttcaattgg<br>atgaagacttaatgatttcagcacaaaaatgggctgaaaatttagccgctgccgaaaaattatatcatagtaattataatgatta<br>tggggaaaatttggcattcaaaatgtctgcatcaccatgtcaaataacaggtgaagaagtttcacagacatggtatagtgagatt<br>gactatcatgactttactcaatcttatcatccaaattcacgtcattttactcagatgatttggaaatctacaacacgagctggat<br>tcggattggcctttagtcaagatcaaacgaaagcatatgttgttggacgttatttacctgtaggtaacaaaggggatttcggatg<br>gaatgtaccacattatcaaggaattaaaagatctgaaagtatgtattcattggatcgtaattcaattagatcaagtcatagacga<br>tcatttagagaaagtctaggattatcagaaagatgcaaatgttactgggatcatacaccatatgcacctgaaggacactctttaa<br>taaacgaagatgaacgctctagaactggttcaatacgtagtggagattttcaacgttccagacctacttcattttcaaataatga<br>tgatatacattataaatctgtatatacaaaaagttcagaagatttacgacctaaaactattaatgtcactactactagtttaccg<br>aatacagatgatgcacataattcattatgtagtccaacagtgatcaatttaaattttatgaattcagatgaacgtccaaatgact<br>tttctggatccagtcgtatgtctattagaacatcagtaagacgcccaacaagaagttcaacaccagtgtgtaatggacactcata<br>tgaaagagagagtagtatttcttcaaatggaagagctaactatctaaaagatgatcaatatggaagagctagttatatcagaaat<br>gatcaatctggaagatctagttatatgaaagatgaacaatatggaaatgcaaatgatgataatagtagtacattaaggggttcga<br>gtgtttctggcaaaggatacaatattgttcatactgtaaacaacgtggacataacaggtatatttcgagaatgtgagacaatat<br>gagtgggttgttattgaaagagattatagaagatattga
SmVAL7	tatagatggtatacacaaaactctgaattattagctttacacaatgcatatagaaggaatataaaatatggtaatgttcgtgatc<br>aaccacaagtgatgagtatgcttaaactaacatggagtcataaattggctgaaatggctcaggaatgggcattacaatgtgtgcc<br>aagacgaagtaacatgactatgaggaaaggttcgaaatggacatatgttggtcagagtatcgctttcgttcctaaagttagacaa<br>gcagcatctgtatggtttgaacaacacaagaattacaatttcgaaaacaatacttgtgaggcaaataaaacttgtgcagattaca<br>aacaattagctttcgccgataccacacatattggatgtggttatgcaatgtgttttaatctaacaggactagataaagtttttgt<br>tgtttgcaactatggaccagggggtaaatatgccaatagacaaccctatgatcctatatatcctgaagatccatactacctccca<br>tga
SmVAL13	atggtagatgaacaattaaatcatgatgcattgaatgaacataatcgattacgtgcattgcacggatgtcctccattaaaatatg<br>atcgaagacttgctagagaagcacaagcttgggctgaaaatcttgcacgtttaaaaattatggaacatagtatctgtgatgaata<br>tggagaaaatttggcatcagcacaatctactggaaaagcggaaatgaccggagctagagcgacacgaaactggtacgatgagatt<br>cattatcacaattttaataaacagtttcaaagtcagtcaggtcattttacacagcttatctggaagaatacatcaaaagcaggat<br>ttggcattcagcattcagttgatggccatcatgtttttatagtaggacgttatgagccacctgggaatgtaaatggtcaattttt<br>agaaaatgtacctccacctatccatggacagtcaactcccaaatctaaagtcccttcttacaaacataatgaacagaatggtcca<br>cgaagaacctatcaagatgaactagttattgttcgagagacagatagaaaagatcacaatggttcaaatcatattacgttgatag

	atagttctaaacgaagcaggtcagatgaaactataccaaacaaaaacgaagtacgtatcattagagcggagaagaaacgtcaacg<br>aagatgtgcaaagaggtgtagtattatgtaa
SmVAL16	atgataataaataaacttaataaggaggctatacaggctcataatgaacttagagcgttacatggttgtccagaaataagttatg<br>attcaaaactggctagtgattctcaaaagtgggctgaacatttggcttcaataaattgtttacaacatagtaaaggagatgatta<br>tggtgaaaatttagcatttcaaatgtctacagccggtgcatcattgaatggtcgtgaagcaactcgaaattggtacgatgaaatt<br>attcaacatgattttaatggacaaaatcaacctgggacaggacattttactcaagtaatttggaagtcaactattaaagcaggat<br>tcggttctgcattatccaaa
SmVAL24	ttggacccacaggaggcacaactcatgctgcgagtacataatgagcatcgagcataccgaaaactgtgtggcgaagaggacatag<br>tgccagcagaggaaatactacagcctcttgagtgggacgataaattggctgccgctgctcaaagttggtctgaaaaatgcaatcc<br>atttgacgaagaaccgattggaaatgttggtaaatgggattcggttggtcgaaattccgctattcattctgagttggcagaagct<br>gttgcatattggatgaaagaatcgaactactatgaccacaattcagacctctgtgagccatcgcatcactgcaacacttacaagc<br>agatagttgaagcacaaacggcgtacgtgggatgcggttataccagatgtgaagaatatgaatatccatcaaacatgttgatcgc<br>ttgttactactcacctaaagtgatgagtggtccaccgtacactgacggaacgaacggacgatgcggatctgaatga
